# Draft Genome Sequence of an Industrially Important *Bacillus* sp. from Mandarmani Coastal Waters in Midnapur District, West Bengal, India

**DOI:** 10.1128/genomeA.00867-16

**Published:** 2016-08-18

**Authors:** Shaon Ray Chaudhuri

**Affiliations:** a Department of Microbiology, Tripura University, Suryamaninagar, Tripura West, India; b Centre of Excellence in Environmental Technology and Management, Maulana Abul Kalam Azad University of Technology, West Bengal, Kolkata, India

## Abstract

Reported here is the draft genome sequence of an amylase-, protease-, DNase-, oxidase-, gelatinase-, and catalase-producing, Gram-positive diplobacillus (*Bacillus* sp. SM1 strain MCC2138), which was isolated from marine coastal waters and has the ability to degum raw silk fabric as well as Ramie fiber. The genome comprises 1.76 Mb with a GC content of 34.5%.

## GENOME ANNOUNCEMENT

The marine environment is rich in microbial diversity with an expected population of about 3.67 × 10^28^ microorganisms (1). This environment shows striking differences in terms of pressure, salinity, temperature, and light from other water bodies and is expected to harbor diverse microbial populations belonging to aerobic, anoxygenic, and phototrophic groups simultaneously. These populations demonstrate adaptability to maintain intracellular sodium ion concentrations and hence avoid osmotic shock (2). They are a promising source for natural products of the future due to the incredible diversity of chemical compounds that have been isolated from them (3). Proteases account for 60% of the world enzyme market, while 40% are of microbial origin (4–6). In lieu of the wide applications of proteases, this biofilm-forming Gram-positive diplobacillus, with optimum temperature of 37°C and pH of 7, was isolated from Mandarmani coastal waters in milk media. The strain (MCC2138) was closest to *Bacillus thuringensis* at the 16SrDNA (FJ377720) sequence level. It could accumulate Pb, Cr, Ni, Cu, and Co (7). Its extracellular enzyme was efficient in degumming of raw silk fabric (8) as well as Ramie fiber (9).

SOAPdenovo version 2.04-r240 was used to assemble the raw reads (29 contigs) with a 2.0× coverage generated on an Illumina platform, totaling 17,69,494 bp, with a largest contig length of 5,01,909 bp and *N*_50_ and *L*_50_ values of 216,057 bp and 3 bp, respectively. The contigs submitted to the RAST server (10) revealed 5,580 genes, of which only 27% fall under the subsystem category. The maximum similarity was with *Bacillus cereus* followed by *Bacillus* sp. and *Bacillus thuringiensis*, as per RAST analysis. The functional annotation using Blast2GOPro version 2.8 revealed 3,380 genes responsible for biological processes, 2,210 for cellular components, and 3,205 for molecular functions. A total of 2,059 genes were annotated for metabolic pathways.

The important genes contained in the different contigs are as follows: ABC transporter ATP binding protein; redox-sensitive transcriptional regulator (AT-rich DNA binding protein); heat shock protein 60 family cochaperone GroES/GroEL; ATP pyrophosphatase subunit (EC 6.3.5.2); phosphate regulon transcriptional regulatory protein PhoB (SphR) (accession no. LXJX01000001.1); oligopeptide ABC transporter, periplasmic oligopeptide-binding protein OppA/B/C/D/F (TC3.A.1.5.1); hydrolase, tRNA; glycerate kinase (EC2.7.1.31); arginase (EC3.5.3.1); diadenylate cyclase spyDAC; bacterial checkpoint controller DisA with nucleotide-binding domain; phosphoglucosamine mutase (EC 5.4.2.10; accession no. NZ_LXJX01000003.1); programmed cell death toxin YdcE and YdcD; outer membrane lipoprotein receptor; Holo (acyl carrier protein) synthase (EC2.7.8.7); UV endonuclease UvdE family; DEAD box ATP-dependent RNA helicase CshA (EC3.6.4.13); t-RNA (accession no. NZ_LXJX01000007.1); ABC transporter permease protein YvcS; ABC transporter ATP-binding protein YvcR; beta-lactamase class A; secreted and spore coat–associated protein 1; chitinase (EC 3.2.1.14); cell-division protein FtsW; microbial collagenase (EC 3.4.24.3); transcriptional regulator, TetR family (accession no. NZ_LXJX01000017.1); cysteine synthase (EC 2.5.1.47); chaperonin (heat shock protein 33); tRNA (Ile) lysidine synthetase (EC 6.3.4.19); AbrB family transcriptional regulator (SpoVT); transcription repair coupling factor; Fin, required for the switch from sigma F to sigma G during sporulation; peptidyl-tRNA hydrolase (EC 3.1.1.29) (accession no. NZ_LXJX01000019.1).

### Accession number(s).

This whole-genome shotgun project has been deposited at DDBJ/ENA/GenBank under the accession number LXJX00000000. The version described in this paper is the first version, LXJX01000000.
